# The SNPs rs429358 and rs7412 of *APOE* gene are association with cerebral infarction but not SNPs rs2306283 and rs4149056 of *SLCO1B1* gene in southern Chinese Hakka population

**DOI:** 10.1186/s12944-020-01379-4

**Published:** 2020-09-05

**Authors:** Heming Wu, Qingyan Huang, Zhikang Yu, Hailing Wu, Zhixiong Zhong

**Affiliations:** 1 Center for Precision Medicine, Meizhou People’s Hospital (Huangtang Hospital), Meizhou Academy of Medical Sciences, Meizhou Hospital Affiliated to Sun Yat-sen University, Meizhou, P. R. China; 2 Guangdong Provincial Key Laboratory of Precision Medicine and Clinical Translational Research of Hakka Population, Meizhou People’s Hospital (Huangtang Hospital), Meizhou Academy of Medical Sciences, Meizhou Hospital Affiliated to Sun Yat-sen University, Meizhou, P. R. China; 3 Guangdong Provincial Engineering and Technology Research Center for Clinical Molecular Diagnostics and Antibody Therapeutics, Meizhou People’s Hospital (Huangtang Hospital), Meizhou Academy of Medical Sciences, Meizhou Hospital Affiliated to Sun Yat-sen University, Meizhou, P. R. China; 4 Guangdong Provincial Engineering and Technology Research Center for Molecular Diagnostics of Cardiovascular Diseases, Meizhou People’s Hospital (Huangtang Hospital), Meizhou Academy of Medical Sciences, Meizhou Hospital Affiliated to Sun Yat-sen University, Meizhou, P. R. China; 5 Center for Cardiovascular Diseases, Meizhou People’s Hospital (Huangtang Hospital), Meizhou Academy of Medical Sciences, Meizhou Hospital Affiliated to Sun Yat-sen University, Meizhou, P. R. China

**Keywords:** Apolipoprotein E, Solute carrier organic anion transporter family member 1B1, Gene polymorphism, Cerebral infarction, Relationship, Hakka

## Abstract

**Background:**

Apolipoprotein E (ApoE) and solute carrier organic anion transporter family member 1B1 (SLCO1B1) regulate lipid metabolism. However, the relationship between genetic polymorphisms of *APOE* and *SLCO1B1* and cerebral infarction (CI) remains unclear.

**Methods:**

A total of 938 CI patients and 1028 control participants were included in the study. The rs429358 and rs7412 single nucleotide polymorphisms (SNPs) in the *APOE* gene and rs2306283 and rs4149056 SNPs in the *SLCO1B1* gene were analyzed by fluorescence polymerase chain reaction (PCR).

**Results:**

The genotype ɛ3/ɛ3 was the most common *APOE* genotype, with ɛ3 being the allele with the highest frequency, followed by ɛ4 and ɛ2. Statistically significant differences of genotype ɛ2/ɛ2 (χ^2^ = 3.866, *P* = 0.049), ɛ2/ɛ3 (χ^2^ = 20.030, *P* < 0.001), ɛ3/ɛ4 (χ^2^ = 16.960, *P* < 0.001), and ɛ4/ɛ4 (χ^2^ = 4.786, *P* = 0.029) between CI patients and controls were detected. The *SLCO1B1* genotype *1b/*1b and haplotype *1b showed the highest frequency in the study sample. There was no statistically significant difference in the frequencies of *SLCO1B1* genotypes and haplotypes among CI patients comparing with controls. Moreover, ε4 carriers had significantly higher low-density lipoprotein-cholesterol (LDL-C) and apolipoprotein B (Apo-B) and lower apolipoprotein A1 (Apo-A1)/Apo-B levels than ε2 and ε3 carriers, but ε2 carriers showed lower LDL-C and Apo-B and higher Apo-A1/Apo-B than ε3 and ε4 carriers. Further, logistic regression analysis revealed that high LDL-C, high ApoB, smoking, hypertension and the ε4 allele were risks for the presence of CI.

**Conclusions:**

This study indicated that the *APOE* SNPs rs429358 and rs7412 may be associated with susceptibility to cerebral infarction in southern Chinese Hakka population.

## Introduction

Ischemic cerebral infarction (CI) refers to ischemic necrosis or softener of local brain tissue caused by cerebral blood circulation disorder, ischemia and hypoxia, with appearance of corresponding neurological defects. Clinically, patients often have vertigo, diplopia, gait instability, limb shaking and other manifestations. CI is a major disease that seriously endangers human health, accounting for approximately 70% of stroke [1]. The risk of CI may vary according to the presence of various genetic and environmental factors. In addition, the relationship between the functional variation of multiple gene in different ethnic groups and CI risk has been studied, including the polymorphisms of interleukin-6 gene [2], β-fibrinogen gene [3], PON1 hypermethylation and PON3 hypomethylation [4], 5-lipoxygenase-activating protein gene association (ALOX5AP) [5], the cytochrome P450 1A1 (CYP1A1) gene [6], the human scavenger receptor class B type I (SR-BI) gene [7], genes associated with lipid metabolism [8, 9], and others [10]. In general, it is important for both risk prediction and prevention of CI by identifying genetic risk.

Apolipoprotein E (ApoE), a polymorphic protein that is mainly synthesized by the liver, can bind with chylomicron, high-density lipoprotein-cholesterol (HDL-C), low-density lipoprotein-cholesterol (LDL-C), and very low-density lipoprotein-cholesterol (VLDL-C) to participate in the transformation and metabolism of lipoprotein. ApoE plays an important role in regulating lipid metabolism by regulating the bind of these lipoproteins to specific receptors. ApoE is encoded by the *APOE* gene (OMIM 107741), which is located on chromosome 19. There are two common single-nucleotide polymorphisms (SNPs) of the *APOE* gene: 388 T > C (rs429358) and 526C > T (rs7412). Three haplotypes (ɛ2(388 T–526 T), ɛ3(388 T-526C), ɛ4(388C-526C)) and six genotypes (ɛ2/ɛ2, ɛ2/ɛ3, ɛ2/ɛ4, ɛ3/ɛ3, ɛ3/ɛ4, ɛ4/ɛ4) can be formed by these SNPs [11]. Compared with ɛ3 homozygotes, patients with the ɛ2 allele have lower circulating total cholesterol (TC) levels and higher triglyceride levels, whereas those who carry the ɛ4 allele appear to have higher plasma levels of TC and LDL-C [12].

Organic anion transporter family member 1B1 (OATP1B1, also known as solute carrier organic anion transporter family member 1B1 (SLCO1B1)) is a type of intake transporter with specific expression in liver and is responsible for the transport of endogenous and exogenous substances. OATP1B1 is encoded by the *SLCO1B1* gene (OMIM 604843), which is located on chromosome 12p12.1 and is approximately 11 kb in length. The two common SNPs in the *SLCO1B1* gene are 388A > G (rs2306283) and 521 T > C (rs4149056) [13, 14]. These two SNPs can be combined to produce four different haplotypes: *1a (388A-521 T), *1b (388G-521 T), *5 (388A-521C) and *15 (388G-521C) [15–17]. Most studies to date on *SLCO1B1* have focused on the effect of *SLCO1B1* polymorphisms on the pharmacokinetics, efficacy and side effects of oral hypoglycemic agents, statins, and antitumor agents [18, 19].

However, there are few studies on the correlation between *SLCO1B1* polymorphisms and CI. Although some studies in China and elsewhere have used the *APOE* gene as a candidate gene to analyze cardiovascular and cerebrovascular diseases, results in different regions and populations may be inconsistent. In the present study, *SLCO1B1* and *APOE* allele/genotype frequencies and the correlation between *SLCO1B1* and *APOE* polymorphisms and CI were analyzed.

## Materials and methods

### Population samples

**Fig. 1:**
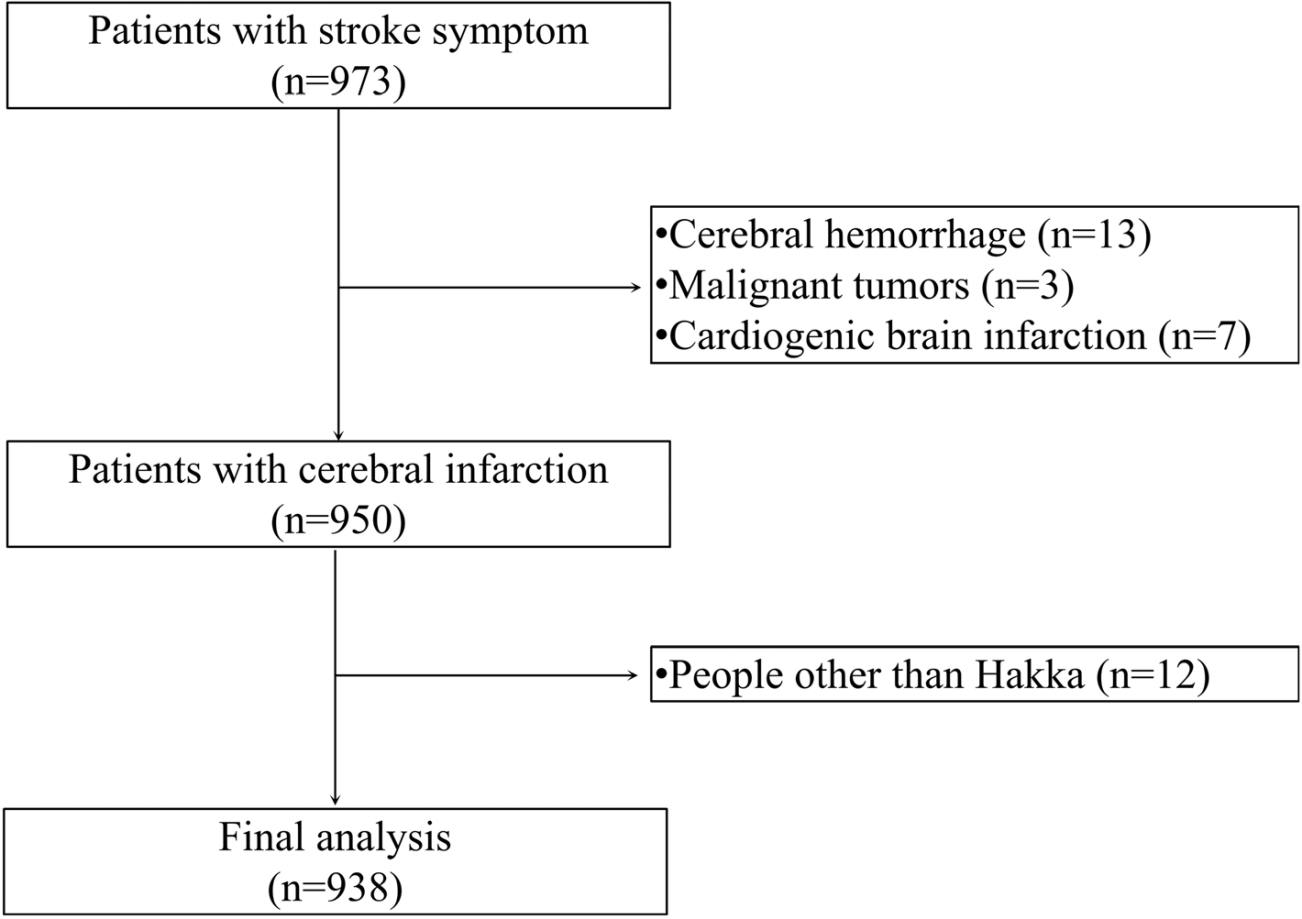
Flow diagram of the study population recruitment process

A total of 1966 individuals were recruited from the inpatients of Meizhou People’s Hospital (Huangtang Hospital), Guangdong province, China, between September 2016 and December 2018; the sample consisted of 938 ischemic cerebral infarction patients and 1028 individuals with non-cerebral infarction as controls. CI patients’ diagnoses were verified by neurologists according to clinical symptoms and computed tomography (CT)/magnetic resonance imaging (MRI). Patients with transient ischemic attacks, cardiogenic cerebral infarctions, cerebral hemorrhage, or malignant tumors were excluded. Information recorded included age, sex, and cerebrovascular disease risk factors. A flow diagram of the study population recruitment process is illustrated in Fig. 1. Patients were recruited after being diagnosed with CI and with the consent of the patients or their family members. All control subjects were randomly selected from the Physical Examination Center of the Meizhou People’s Hospital during the same period. This retrospective case control study was approved by the Human Ethics Committees of Meizhou People’s Hospital (Clearance No.: 2016-A-29).

### Plasma lipid measurements

On the second day of admission, approximately 3 ml of blood was taken from each subject, and plasma was isolated and tested promptly or store at − 80 °C for further analysis. Serum samples were evaluated using the Olympus AU5400 system (Olympus Corporation, Tokyo, Japan) for TC, triglyceride (TG), LDL-C, HDL-C, apolipoprotein B (Apo-B) and apolipoprotein A1 (Apo-A1). TC, TG, LDL-C, HDL-C, Apo-A1/Apo-B analyses were carried out using cholesterol esterase/peroxidase (CHOD/PAP) enzymatic method, Glycerophosphate oxidase/peroxidase (GPO-PAP) enzymatic method, direct surfactant removal method, direct immunoinhibition method, and immunoturbidimetry method, respectively.

### DNA extraction and genotyping assay

Genomic DNA was extracted from whole blood in EDTA using a QIAamp DNA Blood Mini Kit (Qiagen GmbH, North Rhine-Westphalia, Germany) according to the protocol provided. The DNA concentration was measured using a Nanodrop 2000™ Spectrophotometer (ThermoFisher Scientific, Massachusetts, USA). TaqMan probe fluorescent PCR was used for *SLCO1B1* and *APOE* genotyping, in which different primers and probes combinations were designed for different SNP polymorphisms of the *SLCO1B1* and *APOE* genes, and gene polymorphisms at different loci were detected through different channels in the reaction system. Polymerase chain reaction (PCR) was used to amplify the target fragments ((step 1: 37°C for 10 min; step 2: 95°C for 5 min; step 3 (amplification of 40 cycles): 95 °C for 15 s and 60°C for 1 min)). The fluorescence signals, including FAM (SLCO1B1*1b 388A, SLCO1B1*5521 T, ApoE2 526C, ApoE4 388 T), VIC (SLCO1B1*1b 388G, SLCO1B1*5521C, ApoE2 526 T, ApoE4 388C) and ROX (internal standard) (Youzhiyou Medical Technology Co.,Ltd., Hubei, China) were collected using a Roche LightCycler 480 II.

### Statistical analysis

Data analysis was performed using SPSS statistical software version 21.0 (IBM Inc., State of New York, USA). Continuous variable data are represented by mean ± standard deviation (SD), and categorical variables are represented by percentages. Student’s t test or the Mann-Whitney U test was used for continuous data analysis. Genotype composition ratios and allele frequencies between groups were analyzed by the chi-square test. Logistic regression analysis was applied to assess the interactions between *SLCO1B1* and *APOE* polymorphisms and various factors (smoking, alcohol, hypertension, etc) in cerebral infarction. *P* < 0.05 was considered statistically significant.

## Results

### Population characteristics

The 1966 individuals in this study, with ages between 20 and 99 years, consisted of 938 CI patients (581 males and 357 females) and 1028 individuals with non-CI (622 males and 406 females) as controls. As shown in Table 1, the CI patients’ average age was 65.6 ± 10.6 years, with 63.9 ± 9.9 years for males and 68.3 ± 11.2 years for females. The average age of the controls was 63.7 ± 12.4 years, with 62.9 ± 12.6 years and 64.8 ± 12.1 years for males and females, respectively. There were statistically significant differences in percentage of smokers (*P* < 0.001), prevalence of hypertension (*P* < 0.001) and TG level (*P* < 0.001) between the patients and controls, though there were no statistically significant differences in age, TC, HDL-C, LDL-C, Apo-A1, Apo-B, Apo-A1/Apo-B or percentage of alcohol cases. The differences in prevalence of hypertension (*P* < 0.001) and TG levels (*P* = 0.003) between female patients and female controls were also statistically significant, and prevalence of hypertension (*P* < 0.001) and TG levels (*P* = 0.009) were significant among males.

**Table 1:** Clinical characteristics of males and females in cerebral infarction patients and control participants

	Total (*n* = 1966)			Male (*n* = 1203)			Female (*n* = 763)		
	Patient Group	Control Group	*P* values	Patient Group	Control Group	*P* values	Patient Group	Control Group	*P* values
No. of subjects	938	1028		581	622		357	406	
Age, y	65.5 ± 10.6	63.7 ± 12.4	0.050	63.9 ± 9.9	62.9 ± 12.6	0.157	68.3 ± 11.2	64.8 ± 12.1	< 0.001
Smokers, n(%)	258 (27.5%)	224 (21.8%)	0.003	256 (44.1%)	224 (36.0%)	0.004	2 (0.6%)	0 (0)	0.219
Alcohol, n(%)	40 (4.3%)	39 (3.8%)	0.596	40 (6.9%)	39 (6.3%)	0.667	0 (0)	0 (0)	–
Hypertension, n(%)	663 (70.7%)	505 (49.1%)	< 0.001	406 (69.9%)	287 (46.1%)	< 0.001	257 (72.0%)	218 (53.7%)	< 0.001
TG, mmol/L	2.145 ± 2.388	1.798 ± 1.443	< 0.001	2.128 ± 2.560	1.800 ± 1.608	0.009	2.171 ± 2.101	1.795 ± 1.126	0.003
TC, mmol/L	5.114 ± 1.398	5.047 ± 1.281	0.268	4.967 ± 1.280	5.009 ± 1.450	0.595	5.276 ± 1.299	5.178 ± 1.273	0.294
HDL-C, mmol/L	1.279 ± 0.373	1.271 ± 0.330	0.623	1.231 ± 0.358	1.219 ± 0.293	0.533	1.355 ± 0.368	1.352 ± 0.384	0.907
LDL-C, mmol/L	2.924 ± 0.902	2.864 ± 0.948	0.157	2.902 ± 0.909	2.836 ± 0.957	0.221	2.959 ± 0.890	2.908 ± 0.935	0.441
Apo-A1, g/L	1.142 ± 0.291	1.153 ± 0.333	0.425	1.096 ± 0.260	1.098 ± 0.299	0.905	1.215 ± 0.322	1.237 ± 0.364	0.396
Apo-B, g/L	0.904 ± 0.266	0.903 ± 0.298	0.909	0.899 ± 0.268	0.890 ± 0.302	0.582	0.912 ± 0.264	0.922 ± 0.292	0.631
Apo-A1/ Apo-B	1.359 ± 0.512	1.404 ± 0.611	0.079	1.316 ± 0.498	1.370 ± 0.626	0.097	1.429 ± 0.527	1.455 ± 0.584	0.514

Values for age expressed as mean ± SD

*TG* triglycerides

*TC* total cholesterol

*HDL-C* high-density lipoprotein-cholesterol

*LDL-C* low-density lipoprotein-cholesterol

*Apo-A1* apolipoprotein A1

*Apo-B* apolipoprotein B

### Genotype and haplotype frequencies of *APOE* gene

Among all subjects, the frequencies of genotypes ɛ3/ɛ3, ɛ3/ɛ4, ɛ2/ɛ3, ɛ2/ɛ4, ɛ4/ɛ4, and ɛ2/ɛ2 were 73.60, 13.33, 9.87, 1.58, 1.07, and 0.56%, respectively. The frequencies of alleles ɛ3, ɛ4 and ɛ2 were 85.20, 8.52, and 6.28% respectively. The genotype distributions in both the CI patients and control participants were consistent with Hardy-Weinberg equilibrium (χ^2^ = 4.495, *P* = 0.488 and χ^2^ = 1.855, *P* = 0.879, respectively). As the results showed, ɛ3/ɛ3 was the most common *APOE* genotype, and ɛ3 was the allele with the highest frequency, followed by ɛ4 and ɛ2 (Table 2).

**Table 2:** Genotypes and allele distribution of *APOE* gene in cerebral infarction patients and control participants in total and according to gender

**Genotypes**	**ɛ2/ɛ2**	**ɛ2/ɛ3**	**ɛ2/ɛ4**	**ɛ3/ɛ3**	**ɛ3/ɛ4**	**ɛ4/ɛ4**
All subjects	11 (0.56%)	194 (9.87%)	31 (1.58%)	1447 (73.60%)	262 (13.33%)	21 (1.07%)
Patients(*n* = 938)	2 (0.21%)	63 (6.72%)	18 (1.92%)	684 (72.92%)	156 (16.63%)	15 (1.60%)
Controls(*n* = 1028)	9 (0.88%)	131 (12.74%)	13 (1.26%)	763 (74.22%)	106 (10.31%)	6 (0.58%)
*P* values (Patients vs controls)	0.049	< 0.001	0.279	0.513	< 0.001	0.029
Males						
Patients(*n* = 581)	2 (0.34%)	41 (7.06%)	16 (2.75%)	409 (70.40%)	104 (17.90%)	9 (1.55%)
Controls(*n* = 622)	4 (0.64%)	86 (13.83%)	8 (1.29%)	458 (73.63%)	63 (10.13%)	3 (0.48%)
*P* values (Patients vs controls)	–	< 0.001	0.069	0.211	< 0.001	–
Females						
Patients(*n* = 357)	0 (0)	22 (6.16%)	2 (0.56%)	275 (77.03%)	52 (14.57%)	6 (1.68%)
Controls(*n* = 406)	5 (1.23%)	45 (11.08%)	5 (1.23%)	305 (75.12%)	43 (10.59%)	3 (0.74%)
*P* values (Patients vs controls)	–	0.017	0.458	0.538	0.097	–
**Alleles**	**ɛ2**	**ɛ3**	**ɛ4**			
All subjects(*n* = 3932)	247 (6.28%)	3350 (85.20%)	335 (8.52%)			
Patients(*n* = 1876)	85 (4.53%)	1587 (84.59%)	204 (10.87%)			
Controls(*n* = 2056)	162 (7.88%)	1763 (85.75%)	131 (6.37%)			
*P* values (Patients vs controls)	< 0.001	0.309	< 0.001			
Males						
Patients(*n* = 1162)	61 (5.25%)	963 (82.87%)	138 (11.88%)			
Controls(*n* = 1244)	102 (8.20%)	1065 (85.61%)	77 (6.19%)			
*P* values (Patients vs controls)	0.004	0.065	< 0.001			
Females						
Patients(*n* = 714)	24 (3.36%)	624 (87.39%)	66 (9.24%)			
Controls(*n* = 812)	60 (7.39%)	698 (85.96%)	54 (6.65%)			
*P* values (Patients vs controls)	0.001	0.411	0.060			

Numbers in parentheses are percentages

There were statistically significant differences in genotype ɛ2/ɛ2 (χ^2^ = 3.866, *P* = 0.049), ɛ2/ɛ3 (χ^2^ = 20.030, *P* < 0.001), ɛ3/ɛ4 (χ^2^ = 16.960, *P* < 0.001), and ɛ4/ɛ4 (χ^2^ = 4.786, *P* = 0.029) in the patients compared with the controls. The frequencies of genotypes ɛ2/ɛ3 (χ^2^ = 14.579, *P* < 0.001) and ɛ3/ɛ4 (χ^2^ = 15.177, *P* < 0.001) between male patients and male controls showed statistically significant differences; in contrast, a significant difference only in genotype ɛ2/ɛ3 (χ^2^ = 5.744, *P* = 0.017) was detected among females (Patients vs Controls = 6.16% vs 11.08%). The frequencies of allele ɛ2 (χ^2^ = 18.682, *P* < 0.001) and ɛ4 (χ^2^ = 25.516, *P* < 0.001) showed statistically significant differences in the patients compared with controls, including between male patients and male controls and between female patients and female controls, respectively (Table 2).

### Genotype and haplotype frequencies of *SLCO1B1* gene

The frequencies of genotypes *1b/*1b, *1a/*1b, *1b/*15, *1a/*15, *1a/*1a, *15/*15, and *1a/*5 were 41.40, 32.50, 13.48, 5.95, 5.44, 1.17, and 0.05%, respectively, in all subjects. The corresponding frequencies in the CI patient group were 38.70, 33.69, 13.75, 6.72, 5.86, 1.17, and 0.11%. And 43.87, 31.42, 13.23, 5.25, 5.06, 1.17, and 0% in the control group. The genotype distributions in both the CI patients and control participants were consistent with Hardy-Weinberg equilibrium (χ^2^ = 1.661, *P* = 0.962 and χ^2^ = 0.514, *P* = 0.992, respectively). There was no statistically significant difference in the frequencies of these genotypes in the patients compared with the controls. The frequencies of *SLCO1B1* genotypes between male patients and male controls were not significantly different, nor were those in the female subjects (Table 3).

**Table 3:** Genotypes and allele distribution of *SLCO1B1* gene in cerebral infarction patients and control participants in total and according to gender

**Genotypes**	*15/*15	*1a/*15	*1a/*1a	*1a/*1b	*1a/*5	*1b/*15	*1b/*1b
All subjects	23 (1.17%)	117 (5.95%)	107 (5.44%)	639 (32.50%)	1 (0.05%)	265 (13.48%)	814 (41.40%)
Patients(n = 938)	11 (1.17%)	63 (6.72%)	55 (5.86%)	316 (33.69%)	1 (0.11%)	129 (13.75%)	363 (38.70%)
Controls(n = 1028)	12 (1.17%)	54 (5.25%)	52 (5.06%)	323 (31.42%)	0 (0)	136 (13.23%)	451 (43.87%)
*P* values (Patients vs controls)	0.991	0.171	0.432	0.283	0.477	0.734	0.020
Males							
Patients(n = 581)	8 (1.38%)	40 (6.88%)	35 (6.02%)	199 (34.25%)	1 (0.17%)	79 (13.60%)	219 (37.69%)
Controls(n = 622)	11 (1.77%)	36 (5.79%)	35 (5.63%)	190 (30.55%)	0 (0)	85 (13.67%)	265 (42.60%)
*P* values (Patients vs controls)	0.586	0.435	0.769	0.170	0.483	0.972	0.083
Females							
Patients(n = 357)	3 (0.84%)	23 (6.44%)	20 (5.60%)	117 (32.77%)	0 (0)	50 (14.01%)	144 (40.34%)
Controls(n = 406)	1 (0.25%)	18 (4.43%)	17 (4.19%)	133 (32.76%)	0 (0)	51 (12.56%)	186 (45.81%)
*P* values (Patients vs controls)	–	0.219	0.364	0.997	–	0.557	0.128
**Alleles**	*15	*5	*1a	*1b			
All subjects(n = 3932)	428 (10.89%)	1 (0.03%)	971 (24.69%)	2532 (64.39%)			
Patients(n = 1876)	214 (11.41%)	1 (0.05%)	490 (26.12%)	1171 (62.42%)			
Controls(n = 2056)	214 (10.41%)	0 (0)	481 (23.39%)	1361 (66.20%)			
*P* values (Patients vs controls)	0.315	0.477	0.048	0.014			
Males							
Patients(n = 1162)	135 (11.62%)	1 (0.09%)	310 (26.68%)	716 (61.62%)			
Controls(n = 1244)	143 (11.50%)	0 (0)	296 (23.79%)	805 (64.71%)			
*P* values (Patients vs controls)	0.925	0.483	0.103	0.116			
Females							
Patients(n = 714)	79 (11.06%)	0 (0)	180 (25.21%)	455 (63.73%)			
Controls(n = 812)	71 (8.74%)	0 (0)	185 (22.78%)	556 (68.47%)			
*P* values (Patients vs controls)	0.129	–	0.267	0.051			

Numbers in parentheses are percentages

Four haplotypes of the two SNPs of *SLCO1B1* were analyzed. The *1b (388G-521 T) haplotype (64.39%) presented the highest frequency, followed by haplotype *1a (388A-521 T) (24.69%), *15 (388G-521C) (10.89%) and *5 (388A-521C) (0.03%) haplotypes. The frequencies of *SLCO1B1* haplotypes between male patients and male controls and between female patients and female controls showed no statistically significant differences (Table 3).

### Relationships between serum lipid level and *APOE* allele and logistic regression analysis of the risk of ε4 allele for CI

Relationships between *APOE* alleles (ε2, ε3, and ε4) and serum lipid levels were analyzed. Subjects with the *APOE* ε2/ε4 genotype (*n* = 31) were excluded because ε2 and ε4 alleles play opposing roles in lipid metabolism. In this study, ε4 carriers had significantly higher LDL-C and Apo-B and lower Apo-A1/Apo-B levels than ε2 and ε3 carriers, but ε2 carriers showed lower LDL-C and Apo-B and higher Apo-A1/Apo-B than ε3 and ε4 carriers. There were no significant impacts of *APOE* polymorphism on the TG, TC, HDL-C and Apo-A1 levels (Table 4).

**Table 4:** Relationships between serum lipid level and *APOE* allele in cerebral infarction patients and control participants

Serum lipid level	Cerebral infarction patients (*n* = 920)				Controls (*n* = 1015)			
	ɛ2(*n* = 65)	ɛ3(*n* = 684)	ɛ4(*n* = 171)	*P* values	ɛ2(*n* = 140)	ɛ3(n = 763)	ɛ4(*n* = 112)	*P* values
TG, mmol/L	2.064 ± 1.986	1.787 ± 1.434	1.773 ± 1.285	0.324	2.449 ± 2.917^*^	2.069 ± 2.208	2.310 ± 2.879	0.170
TC, mmol/L	4.893 ± 1.245	5.048 ± 1.272	5.093 ± 1.320	0.560	5.170 ± 1.476	5.096 ± 1.351	5.242 ± 1.558	0.534
HDL-C, mmol/L	1.302 ± 0.336	1.277 ± 0.326	1.234 ± 0.336	0.225	1.281 ± 0.393	1.284 ± 0.367	1.256 ± 0.407	0.762
LDL-C, mmol/L	2.584 ± 0.766^*◇^	2.925 ± 0.894	3.031 ± 0.922	0.003	2.734 ± 0.971^*◇^	2.866 ± 0.917^◇^	3.061 ± 1.064	0.023
Apo-A1, g/L	1.179 ± 0.327	1.150 ± 0.284^◇^	1.098 ± 0.307^*^	0.064	1.174 ± 0.315	1.161 ± 0.344	1.082 ± 0.282	0.050
Apo-B, g/L	0.802 ± 0.222^*◇^	0.905 ± 0.263	0.934 ± 0.276	0.002	0.858 ± 0.312^*◇^	0.904 ± 0.286^◇^	0.955 ± 0.347	0.035
Apo-A1/ Apo-B	1.605 ± 0.739^*◇^	1.366 ± 0.501^◇^	1.244 ± 0.409^*^	< 0.001	1.529 ± 0.643^*◇^	1.401 ± 0.616^◇^	1.260 ± 0.506^*^	0.002

*P* value shows the differences compared between groups (ε2, ε3, ε4)

^*^P < 0.05 versus corresponding ε3 group

^◇^*P* < 0.05 versus corresponding ε4 group

Logistic regression analysis was performed to determine independent predictors for CI (Table 5), and the results indicated significantly higher risks of CI in the presence of high LDL-C (OR 1.524, 95% CI 1.092–2.100, *P* = 0.013), and high ApoB (OR 3.046, 95% CI 1.188–7.809, *P* = 0.020), smoking (OR 1.459, 95% CI 1.166–1.825, *P* = 0.001), hypertension (OR 2.599, 95% CI 2.136–3.164, *P* < 0.001), and the ε4 allele (OR 1.822, 95% CI 1.390–2.388, *P* < 0.001).

**Table 5:** Logistic regression analysis of risks of cerebral infarction in southern Chinese Hakka population

Variables	β	*P* value	Adjusted OR (95% CI)
LDL-C	0.415	0.013	1.092–2.100
ApoB	1.114	0.020	1.188–7.809
Smoking	0.378	0.001	1.166–1.825
Hypertension	0.955	< 0.001	2.136–3.164
ε4 carrier	0.600	< 0.001	1.390–2.388

*OR* odds ratio, *CI* confidence interval, *LDL-C* low-density lipoprotein-cholesterol

## Discussion

Stroke is one of the most common causes of death and long-term disability worldwide [20]. Moreover, the stroke burden in China is expected to increase further due to an aging population and a continuing high prevalence of risk factors such as hyperlipidemia [21]. Cerebral infarction, the most common type of stroke, is known as localized brain tissue necrosis or cerebral ischemia caused by cerebral blood disorders, resulting from a blockage of the blood vessels that supply blood to the brain [22–25]. Many studies have shown that the etiology of cerebral infarction is complex, including genetic and environmental factors [26, 27]. The relationship between genetic polymorphisms of *APOE* and *SLCO1B1* and CI were analyzed in this study.

The *APOE* gene encodes a major lipid-binding protein that serves as a cholesterol carrier [28]. Atherosclerosis is an important pathophysiological basis of CI. *APOE* gene polymorphisms have been shown to be associated with atherosclerosis [29, 30]. However, the results of previous studies on the relationship between *APOE* gene polymorphisms and CI are not very consistent. For example, Yan et al. [31] indicated that the ε4 allele was associated with TC and LDL-C and believed that ε4 was a genetic marker of CI. Liu et al. [32] confirmed by MRI scanning that brain injury was aggravated in ε4 allele carriers CI patients after stroke. However, Wang et al. [33] proposed that *APOE* gene polymorphism did not correlate significantly with CI. Based on the results of the present study, there were statistically significant differences in genotypes ɛ2/ɛ2, ɛ2/ɛ3, ɛ3/ɛ4, and ɛ4/ɛ4 among CI patients compared with controls. ε4 carriers had significantly higher LDL-C and Apo-B and lower Apo-A1/Apo-B levels than the other groups, while ε2 carriers showed lower LDL-C and Apo-B and higher Apo-A1/Apo-B. Logistic regression analysis indicated that participants with ε4 allele had a significantly higher risk of CI.

*APOE* gene polymorphisms are important determinants of blood lipid levels, and it may be the reason for the correlation between *APOE* gene polymorphisms and CI [34]. The *APOE* ε4 allele is associated with higher serum lipid levels, whereas the ε2 allele is associated with the lower levels [35]. In addition, the presence of ε2 has been associated with lower LDL-C level but with no influence on HDL-C level [34]. Another study showed that the C allele of SNP rs2910164 of the miR-146a gene is a risk factor for atherosclerotic cerebral infarction (ACI) and that *APOE* ε4 may enhance ACI susceptibility by reducing miR-146a expression [36].

Furthermore, this study found ɛ3 to be the most common allele of the *APOE* gene, accounting for 85.20%, which was consistent with most previous studies [37, 38]. This indicates that the *APOE* allele frequencies in the Meizhou area are similar to those of the Chinese-Northeast [39], Chinese-Jiangsu Han [40] and Chinese-Kunming Han [41] areas, though the ɛ4 allele frequency in Meizhou is lower than that in Shanghai [42].

The frequencies of *SLCO1B1* genotypes *1b/*1b, *1a/*1b, *1b/*15, *1a/*15, *1a/*1a, *15/*15, and *1a/*5 were 41.10, 32.79, 13.03, 5.93, 5.88, 1.22, and 0.05% respectively. The frequency of *SLCO1B1* haplotype *1b revealed its predominance, accounting for 64.01%, followed by *1a (25.27%), *15 (10.69%) and *5 (0.03%). These results are in agreement with previous studies [43–46]. Greek, German, Indian (North) and Macedonian populations exhibit relatively lower rates of *1b (less than 50%), whereas Thailand and Chinese populations show higher rates, generally above 60–70%. In contrast, the allele frequency of haplotypes *15 and *5 displayed little difference. In this study, there were no statistically significant differences in the frequencies of *SLCO1B1* genotypes and haplotypes between CI patients and control participants. In addition, no information has been published about relationship between cerebral infarction and *SLCO1B1* gene polymorphisms in other populations.

### Study strengths and limitations

There are several strengths of this study. This is the first study about the relationship of cerebral infarction and *SLCO1B1* gene polymorphism. The clinical characteristics, lipid levels and *APOE* gene polymorphism indicators were included into the analysis to exclude the influence of related confounding factors on the results. There are some limitations to this study that should be noted. First, CI is a kind of multifactorial diseases caused by genetic and environmental factors. As a retrospective case control analysis, the limitations of the original data included in this study constrained assessment of potential gene-environment interactions. Second, the sample size of this study is not very large, which may lead to some deviations in the results. Therefore, further study with a larger sample size is one of the next tasks. Third, the study was carried out only in Hakka Chinese people, and whether the same is true for other populations needs further investigation.

## Conclusions

In conclusion, the present study suggests that the SNPs rs429358 and rs7412 of the *APOE* gene but not SNPs rs2306283 and rs4149056 of the *SLCO1B1* gene are associated with ischemic cerebral infarction in the southern Chinese Hakka population. Therefore, *APOE* genotyping may be useful to identify individuals at risk of CI and provide guidance for the institution of individualized preventive strategies and therapies for patients.

## Data Availability

All data generated or analyzed during this study are included in this published article.

## Abbreviations

APOE: apolipoprotein E
SLCO1B1: solute carrier organic anion transporter family member 1B1
TG: triglycerides
TC: total cholesterol
HDL-C: high-density lipoprotein-cholesterol
LDL-C: low-density lipoprotein-cholesterol
Apo-A1: apolipoprotein A1
Apo-B: apolipoprotein B
